# Sex and Estrous Cycle Stage Shape Left-Right Asymmetry in Chronic Hippocampal Seizures in Mice

**DOI:** 10.1523/ENEURO.0041-23.2023

**Published:** 2023-06-02

**Authors:** Cathryn A. Cutia, Leanna K. Leverton, Catherine A. Christian-Hinman

**Affiliations:** 1 Neuroscience Program, University of Illinois Urbana-Champaign, Urbana, IL 61801; 2 Department of Molecular and Integrative Physiology, University of Illinois Urbana-Champaign, Urbana, IL 61801; 3 Beckman Institute for Advanced Science and Technology, University of Illinois Urbana-Champaign, Urbana, IL 61801

**Keywords:** epilepsy, hippocampus, laterality, sex differences

## Abstract

Lateralization of hippocampal function is indicated by varied outcomes of patients with neurologic disorders that selectively affect one hemisphere of this structure, such as temporal lobe epilepsy (TLE). The intrahippocampal kainic acid (IHKA) injection model of TLE allows for targeted damage to the left or right hippocampus, enabling systematic comparison of effects of left-right asymmetry on seizure and nonseizure outcomes. Although varying nonseizure phenotypic outcomes based on injection side in dorsal hippocampus were recently evaluated in this model, differences in chronic seizure patterns in left- (IHKA-L) versus right-injected (IHKA-R) IHKA animals have yet to be evaluated. Here, we assessed hippocampal seizure incidence in male and female IHKA-L and IHKA-R mice. Females displayed increased electrographic seizure activity compared with males at both two and four months postinjection. In addition, IHKA-L females showed higher seizure frequency than IHKA-R on diestrus and estrus at two months postinjection, but seizure duration and percent time in seizures were only higher in IHKA-L females on diestrus. These cycle stage-associated changes, however, did not persist to four months postinjection. Furthermore, this lateralized difference in seizure burden was not observed in males. These results indicate for the first time that the side of IHKA injection can shape chronic electrographic seizure burden. Overall, these results demonstrate a female-specific left-right asymmetry in hippocampal function can interact with estrous cycle stage to shape chronic seizures in mice with epilepsy, with implications for neural activity and behavior in both normal and disease states.

## Significance Statement

Seizures in temporal lobe epilepsy (TLE) often originate in the hippocampus, and patient outcomes can depend on whether the seizures initiate in the left or right hippocampus. Although rodent brain function appears less lateralized than in humans, emerging evidence indicates stronger lateralization of hippocampal function in mice than previously thought. Here, we systematically compared chronic epilepsy profiles in mice based on whether left or right hippocampus is the main site of seizure generation. Males did not show a left-right asymmetry in epilepsy severity, but females showed effects of seizure initiation side that varied with estrous cycle stage. These results thus suggest a female-specific lateralization of hippocampal function can interact with the estrous cycle to shape chronic seizures in mice with epilepsy.

## Introduction

The human brain is structurally and functionally lateralized (Gazzaniga, 1995). Although the hippocampus shows structural symmetry, neuroimaging studies suggest distinct functional roles of the two human hippocampi (Maguire and Frith, 2003; Howard et al., 2011). Importantly, several neurologic diseases affect the hippocampus, and the damage inflicted from neurologic disorders such as stroke and epilepsy can be unilaterally localized. Furthermore, patient outcomes can vary based on whether this damage is present in the left or right hippocampus. For example, patients with ischemic stroke injury in the left hippocampus have more apparent memory dysfunction than those with damage in the right (Schaapsmeerders et al., 2015). Additionally, people with epilepsy whose seizures arise from the left hippocampus show a higher degree of cognitive impairment than with right hippocampal seizure foci (Alessio et al., 2006; Addis et al., 2007; Phuong et al., 2021). This variation in outcomes based on the hemisphere containing the unilateral seizure focus may result from underlying functional lateralization in the hippocampus.

Temporal lobe epilepsy (TLE) is a common form of focal epilepsy in which seizures arise from a specific subregion and hemisphere of the temporal lobe, particularly the hippocampus (Engel, 2001). Interestingly, clinical observations in women with TLE suggest that the lateralization of a patient’s seizure focus can impact seizure patterning in relationship to the menstrual cycle. For instance, clinical evidence suggests left-sided seizure foci are associated with higher incidence of seizures that cluster in the few days before and during menstruation, known as perimenstrual catamenial seizures, whereas right-sided seizure foci are associated with noncatamenial patterning of seizures spread across the menstrual cycle (Herzog, 2008; Quigg et al., 2009). Women with catamenial epilepsy are at higher risk for resistance to antiseizure medications (Choi et al., 2020), underscoring the importance of identifying the underlying mechanisms. Furthermore, recent imaging studies have suggested that shifting levels of ovarian hormones across the menstrual cycle can have structural and functional ramifications on the human hippocampus (Taylor et al., 2020; Gloe et al., 2021), although it remains unclear how these effects may interact with seizure focus lateralization to shape seizure patterning in relation to the ovarian cycle.

Recent studies demonstrating lateralization in the rodent hippocampus (Jordan, 2020) support the use of rodent models in investigating the mechanisms that underlie these differences. The intrahippocampal kainic acid (IHKA) mouse model of TLE, which allows for epileptogenic insults to be selectively targeted to the left or right hemisphere, shows neuropathological changes similar to human TLE (Bouilleret et al., 1999; Riban et al., 2002; Gröticke et al., 2008). This model also displays epileptiform discharges and spontaneous recurrent seizures (Bouilleret et al., 1999) that are hallmarks of human TLE (French et al., 1993; Mathern et al., 1995; Rusina et al., 2021). To date, researchers using this model have arbitrarily targeted the left or right hippocampus for KA injection, in the absence of systematic examination of differential outcomes of left and right IHKA injections. In a recent study, however, nonseizure phenotypic outcomes in C57BL/6J females injected with KA in the left or the right dorsal hippocampus were compared (Cutia et al., 2022). It was determined that dentate gyrus granule cell dispersion was altered in an injection site-specific manner (Cutia et al., 2022), indicating lateralized phenotypes at the level of the hippocampus. However, recent work has suggested that granule cell dispersion does not correlate to chronic seizure severity in left-injected IHKA mice (Lisgaras and Scharfman, 2022). Therefore, whether the lateralization effect in granule cell dispersion contributes to functional differences in hippocampal seizure incidence remains unclear. Furthermore, it is unknown whether potential lateralized differences in seizure occurrence are shaped by animal sex and, in females, estrous cycle stage. Here, the hypothesis that the laterality of IHKA injection leads to differential patterning of subsequent spontaneous recurrent seizures, both based on sex and estrous cycle stage, was tested.

## Materials and Methods

### Animals and estrous cycle monitoring

Animal procedures used in this study complied with the ARRIVE guidelines and were approved by the Institutional Animal Care and Use Committee of the University of Illinois Urbana-Champaign. Female and male C57BL/6J mice (#000664, The Jackson Laboratory) were purchased for delivery at six weeks of age. Mice were then housed in a 14/10 h light/dark cycle (lights off at 7 P.M.) and given food and water *ad libitum*. Animals were group-housed (two to five mice per cage) until the time of electrode implantation, after which all mice were housed singly.

Beginning one week after arrival, estrous cycle monitoring in females was performed between 9 and 11 A.M. using a vaginal cytology protocol previously described (Pantier et al., 2019). Female mice were assessed for at least 14 d to verify regular cycles before entering the study and resumed estrous cycle monitoring for the duration of the local field potential (LFP) recording periods. Cycle lengths for each mouse at each recording period were calculated as the average time to progress from one stage of estrus through all other stages to another phase of estrus for all captured cycles (J. Li et al., 2017, 2018). To evaluate the development of differences in seizures over time, mice were recorded and underwent estrous cycle monitoring at both two and four months postinjection (mpi).

### Stereotaxic IHKA and LFP electrode implantation surgeries

All stereotaxic surgeries were conducted under isoflurane anesthesia (2–3%, vaporized in 100% oxygen) with carprofen (0.5 mg/ml) for analgesia. Female mice underwent stereotaxic unilateral injection of KA (Tocris Bioscience; 50 nl of 20 mm prepared in 0.9% sterile saline) on the first day of diestrus following the first estrous cycle monitoring period. Age-matched males (> postnatal day 60) were injected in the same manner. Injections were randomly targeted to the left or right dorsal hippocampal region (relative to bregma: 2.0 mm posterior, 1.5 mm lateral, 1.4 mm ventral to cortical surface) as previously described (J. Li et al., 2017). Age-matched controls were injected with the same volume at the same location with saline. Saline-injected animals (female: left *n* = 5, right *n* = 5; males: left *n* = 3, right *n* = 4) showed no seizures and thus were not included in analyses.

All animals were allowed two weeks to recover from the injection before undergoing a second surgery for LFP electrode implantation. Two twisted bipolar electrodes (P1 Technologies) were implanted into the ipsilateral hippocampus just dorsal and lateral to the injection site (relative to bregma: 2.0 mm posterior, 1.75 mm lateral, 1.25 mm ventral; Armstrong et al., 2013; J. Li et al., 2020; Cutia et al., 2022; J. Li and Christian-Hinman, 2022). Anchor microscrews (J.I. Morris Co) were placed into the skull and stabilized with dental cement (Teets “Cold Cure” Dental Cement). Mice were singly housed after electrode implantation and for the duration of the remaining experimental period.

### LFP recording and analysis

One week after electrode implantation, the mice were tethered to an electrical commutator (P1 Technologies) connected to a Brownlee 440 amplifier (NeuroPhase) with gain set at 1 K. LFP signals were recorded as the local field potential differential between the two electrodes (Armstrong et al., 2013), sampled at 2 kHz and digitized to recorder software written in MATLAB (Armstrong et al., 2013). Mice were recorded at 1, 2, and 4 mpi. The 2 and the 4 mpi recording periods were evaluated in the current study; the LFP data collected at 1 mpi from the female animals in the present study were included in another study (Cutia et al., 2022), and are therefore not shown here. In addition, some female mice (IHKA-R = 2, IHKA-L = 5) included in this study were included in a previous publication (J. Li et al., 2020), but the analysis of the data from these animals conducted in the previous work is distinct from that reported here.

All recordings were analyzed with an automated electrographic seizure analyzer (Zeidler et al., 2018) with the minimal seizure duration set at 5 s and the interictal interval at 6 s. It should be noted that most seizures detected in the IHKA mouse model are electrographic and do not generalize to behavioral convulsive seizures (Bouilleret et al., 1999; Gröticke et al., 2008; Klein et al., 2015).

### Hippocampal granule cell dispersion visualization and quantification

Brains from a randomly selected subset of females and males (8–10 mice per group) were sectioned into 40-μm-thick coronal sections using a freezing microtome (Leica SM 2010R). Every third section from the dorsal hippocampal region was used for histology, with a total of four to six sections evaluated for each mouse. Sections were stained with cresyl violet (Sigma-Aldrich C5042) for 12 min at room temperature, dehydrated using graded ethanol solutions (70–100%), before being cleaned with Xylene and coverslipped with Permount. Images were collected using an Olympus BX43 brightfield microscope with an Infinity 3-6UR Teledyne Lumenera Camera and Infinity Capture software (Lumenera). Dentate gyrus granule cell dispersion ipsilateral to the KA injection was quantified as previously described (Cutia et al., 2022; Lisgaras and Scharfman, 2022).

### Ovarian hormone assays

After completion of LFP recordings at 4 mpi, trunk blood was collected from the female mice at the time of euthanasia. All female mice were euthanized by decapitation on diestrus between 12 and 4 P.M. Trunk blood was stored at 4°C for 24 h, and centrifuged for 15 min at 4°C. Serum was then isolated and stored at −80°C until the time of assay. ELISAs for progesterone (IBL America, IB79105) and estradiol (American Laboratory Products Co, 11-ESTHU-E01) were performed on serum samples using commercial kits according to the manufacturers’ instructions and evaluated using a Bio-Tek 800TS Microplate Absorbance Reader. One serum sample from the IHKA-L and one from the IHKA-R group were excluded from the study based on low quality. One IHKA-R sample was tested for progesterone but not for estradiol because of limited sample volume. Additionally, given limited sample volume, all samples were run in singlet to accommodate multiple hormone assays. Intra-assay coefficient of variation values calculated from controls run in duplicate were 5.1 for estradiol and 6.8 for progesterone.

### Statistical analysis

All statistical comparisons were performed using R software. Comparisons of seizure parameters between male and female IHKA-L and IHKA-R animals were made using two-way ANOVA and Tukey’s *post hoc* tests. Comparisons of seizure parameters between IHKA-L and IHKA-R females within individual estrous cycle stages were made using two-sample *t* tests, or Wilcoxon rank-sum tests depending on the normality of the data. Comparisons of progesterone, estradiol, and the progesterone-to-estradiol (P_4_:E_2_) ratio between IHKA-L and IHKA-R groups were made using Wilcoxon rank-sum tests. Normality was evaluated using Shapiro–Wilks tests, and homogeneity of variance was evaluated using Levene’s tests. Correlations between percent of time in seizures and granule cell dispersion, estrous cycle length, and hormone levels were made using Pearson’s correlations. Information on the estimation statistics used to generate statistically significant results in the study is provided in Table 1.

**Table 1:** Statistical table of significant results

Figure	Graph	Data structure	Test	*p* value	95% confidence interval
Figure 1*B*	IHKA-L males vs females	Normally distributed	Two-way ANOVA, Tukey’s *post hoc* test	0.005	23.96–38.42 and 41.46–57.72
Figure 1*B*	IHKA-R males vs females	Normally distributed	Two-way ANOVA, Tukey’s *post hoc* test	0.005	14.60–41.80 and 33.88–42.66
Figure 1*B*	IHKA-R males vs IHKA-L females	Normally distributed	Two-way ANOVA, Tukey’s *post hoc* test	0.002	14.60–41.80 and 41.46–57.72
Figure 1*C*	IHKA-L males vs females	Normally distributed	Two-way ANOVA, Tukey’s *post hoc* test	0.02	11.94–17.58 and 17.01–21.67
Figure 1*C*	IHKA-R males vs females	Normally distributed	Two-way ANOVA, Tukey’s *post hoc* test	0.02	9.36–17.16 and 15.65–20.51
Figure 1*C*	IHKA-R males vs IHKA-L females	Normally distributed	Two-way ANOVA, Tukey’s *post hoc* test	0.03	9.36–17.16 and 17.01–21.67
Figure 1*D*	IHKA-L males vs females	Normally distributed	Two-way ANOVA, Tukey’s *post hoc* test	0.0007	9.43–16.87 and 20.87–20.71
Figure 1*D*	IHKA-R males vs females	Normally distributed	Two-way ANOVA, Tukey’s *post hoc* test	0.0007	5.03–17.23 and 15.90–23.00
Figure 1*D*	IHKA-R males vs IHKA-L females	Normally distributed	Two-way ANOVA, Tukey’s *post hoc* test	0.0004	5.03–17.23 and 20.87–20.71
Figure 2*B*	IHKA-L males vs females	Normally distributed	Two-way ANOVA, Tukey’s *post hoc* test	0.006	11.16–16.92 and 15.94–20.22
Figure 2*B*	IHKA-R males vs females	Normally distributed	Two-way ANOVA, Tukey’s *post hoc* test	0.006	10.25–17.07 and 16.85–21.25
Figure 2*B*	IHKA-L males vs IHKA-R females	Normally distributed	Two-way ANOVA, Tukey’s *post hoc* test	0.03	10.25–17.07 and 15.94–20.22
Figure 2*C*	IHKA-L males vs females	Normally distributed	Two-way ANOVA, Tukey’s *post hoc* test	0.02	6.58–16.78 and 12.81–20.15
Figure 2*C*	IHKA-R males vs females	Normally distributed	Two-way ANOVA, Tukey’s *post hoc* test	0.02	6.27–13.29 and 14.67–22.19
Figure 3*A*	Diestrus	Normally distributed	Two-sample *t* test	0.05	29.30–44.04 and 39.53–58.65
Figure 3*A*	Estrus	Normally distributed	Two-sample *t* test	0.02	33.63–44.29 and 42.51–57.33
Figure 3*B*	Diestrus	Non-normal distribution	Wilcoxon rank-sum test	0.04	15.13–21.63 and 19.37–27.37
Figure 3*C*	Diestrus	Non-normal distribution	Wilcoxon rank-sum test	0.04	13.77–24.67 and 23.83–37.59

## Results

### Seizure burden is increased in IHKA females compared with males at two months after injection

**Figure 1.**
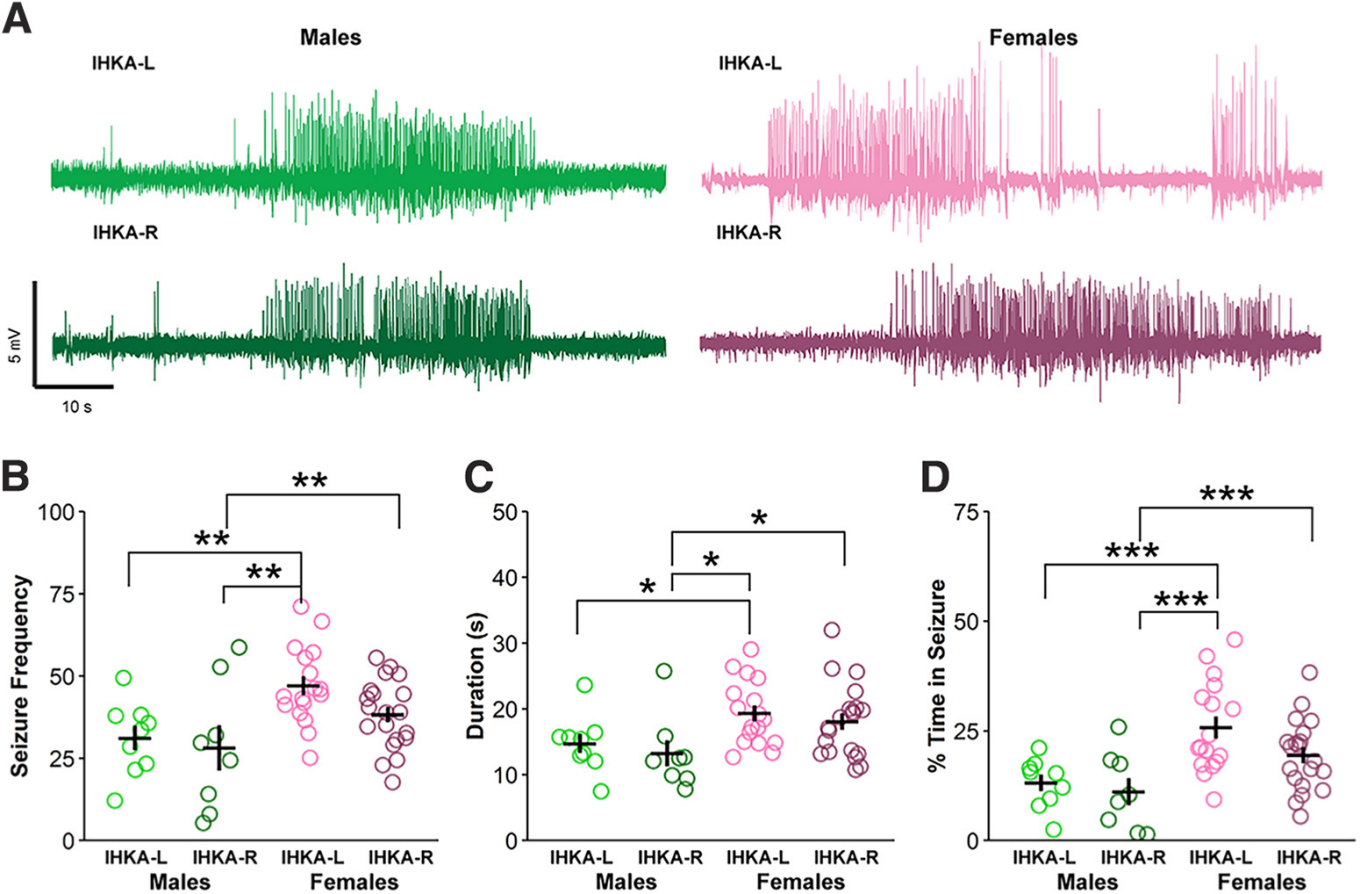
Sex differences in seizure parameters at two months after IHKA injection. ***A***, Example seizure traces. ***B–D***, Circles represent individual values of the average number of seizures per hour (***B***), average seizure duration (***C***), and average percent time in seizure (***D***) for each mouse plotted with mean ± SEM in black lines. *p < 0.05, **p < 0.01, ***p < 0.001 two-way ANOVA and Tukey’s *post hoc* tests.

Both male (IHKA-L = 9, IHKA-R = 8) and female (IHKA-L = 17, IHKA-R = 20) mice were evaluated to characterize sex differences in general seizure patterning and in response to the side of targeted injection (Fig. 1*A*). Recordings collected across a 7-d period at 2 mpi were evaluated for each mouse and averaged for group comparisons. Seizure frequency (seizures per hour) differed between groups based on both injection site (*F*_(1,51)_ = 4.12, *p* = 0.047) and sex (*F*_(1,51)_ = 12.40, *p* = 0.0009; Fig. 1*B*). Specifically, IHKA-L females displayed elevated seizure frequency compared with both IHKA-L (*p* = 0.005) and IHKA-R males (*p* = 0.002; Fig. 1*B*). IHKA-R females also showed higher seizure frequency than IHKA-R males (*p* = 0.005). Seizure duration also displayed a sex difference (*F*_(1,51)_ = 9.79, *p* = 0.002; Fig. 1*C*), with longer seizure duration in IHKA-L females compared with both IHKA-L (*p* = 0.02) and IHKA-R males (*p* = 0.03; Fig. 1*C*). IHKA-R females also showed higher duration compared with IHKA-R males (*p* = 0.02; Fig. 1*C*). The mean percent time in seizures also differed by injection site (*F*_(1,51)_ = 4.45, *p* = 0.04) and sex (*F*_(1,51)_ = 17.14, *p* = 0.0001; Fig. 1*D*), as IHKA-L females spent more time in seizures than IHKA-L (*p* = 0.0007) and IHKA-R males (*p* = 0.0004), and IHKA-R females spent more time in seizures than IHKA-R males (*p* = 0.0007; Fig. 1*D*).

### Sex differences in seizure duration and percent time in seizures persist to four months after injection

**Figure 2.**
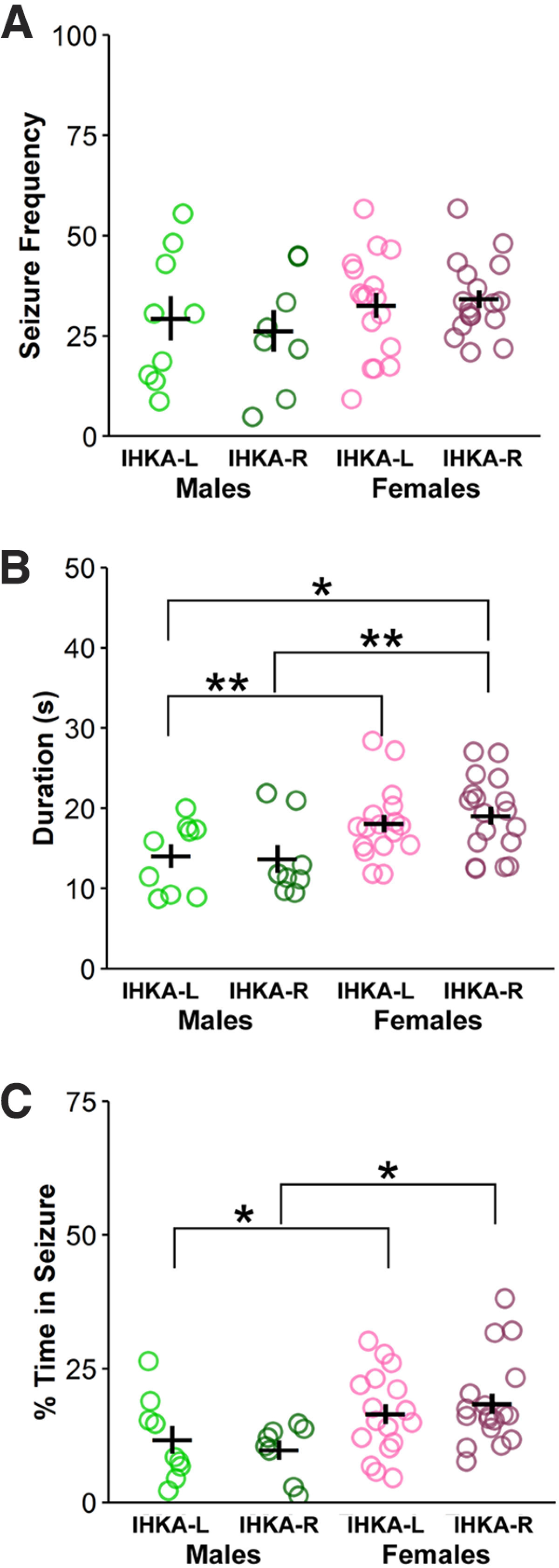
Higher seizure duration in females at four months after IHKA injection. ***A–C***, Circles represent individual values of the average number of seizures per hour (***A***), average seizure duration (***B***), and average percent time in seizure (***C***) for each mouse plotted with mean ± SEM in black lines. *p < 0.05, **p < 0.01 two-way ANOVA and Tukey’s *post hoc* tests.

To characterize the progression of seizure activity over time in these animals, the same animals evaluated at 2 mpi were also evaluated at 4 mpi, with the exception of two IHKA-R females that died before the four-month time point. By 4 mpi, there was no effect of injection site (*F*_(1,49)_ = 0.0001, *p* = 0.99) or sex (*F*_(1,48)_ = 2.17, *p* = 0.15; Fig. 2*A*) on seizure frequency. Seizure duration, however, was still affected by sex and higher in females compared with males (*F*_(1,49)_ = 11.84, *p* = 0.001; Fig. 2*B*). Percent time in seizures was also influenced by sex (*F*_(1,49)_ = 8.95, *p* = 0.004; Fig. 2*B*), as IHKA females displayed higher time in seizures than male counterparts (IHKA-L: *p* = 0.02, IHKA-R: *p* = 0.02; Fig. 2*B*). Together, these results indicate that IHKA females maintain higher seizure duration and percent time in seizures than males when recorded at 4 mpi.

### Left-right asymmetry in seizure burden in IHKA females is most pronounced on diestrus at two months after injection

**Figure 3.**
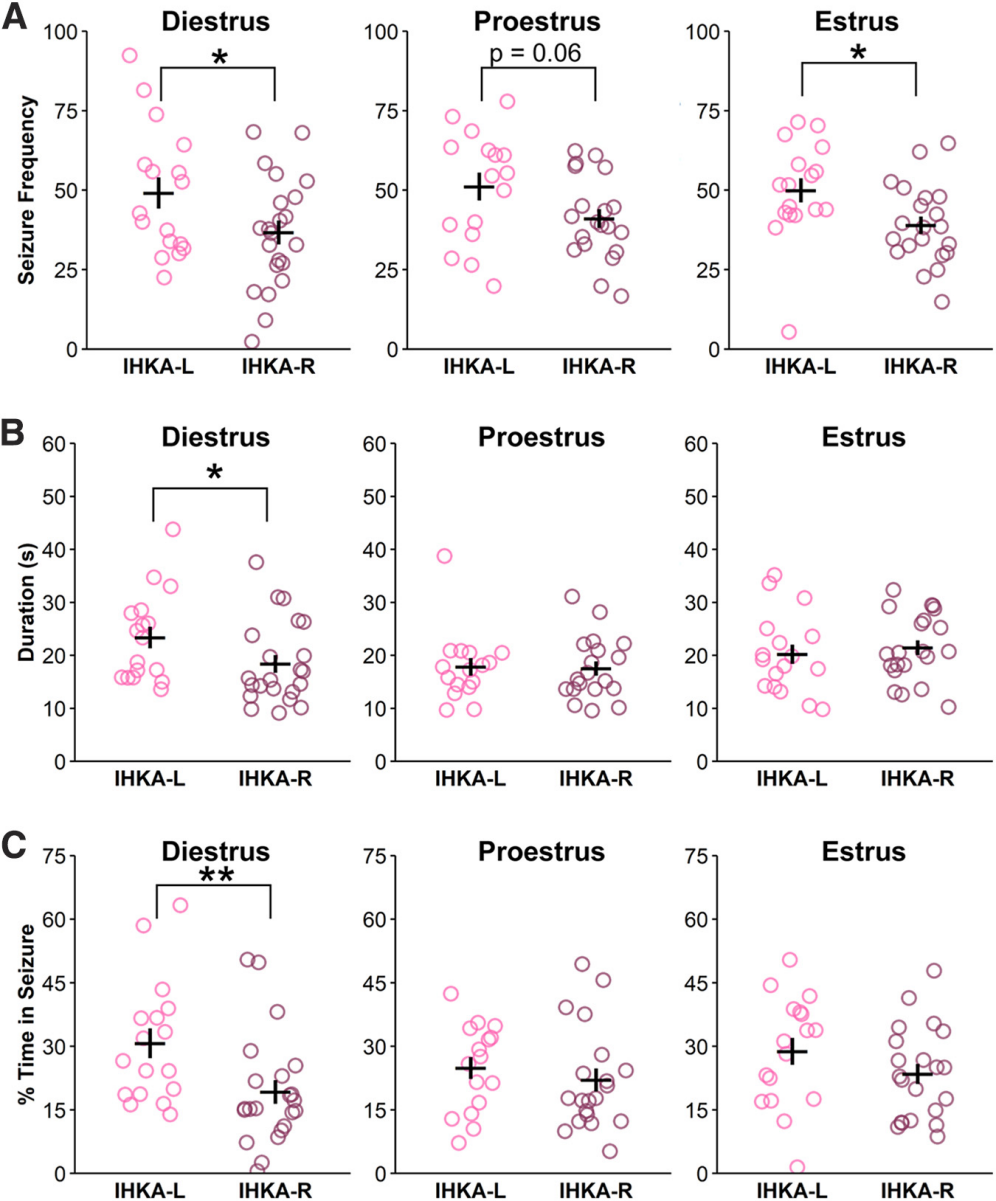
Seizure burden is elevated in IHKA-L females on diestrus two months after IHKA injection. ***A–C***, Circles represent individual values of the average number of seizures per hour (***A***), average seizure duration (***B***), and average percent time in seizure (***C***) for each IHKA female, plotted with mean ± SEM in black lines. *p < 0.05, **p < 0.01 two-sample *t* test or Wilcoxon rank-sum test based on data normality.

To investigate the relationship of estrous cycle phase to seizure patterns, female animals were evaluated across diestrus, estrus, and proestrus. Recordings from 3 d of each estrous cycle stage from at least three different cycles per mouse were averaged to quantify seizure parameters present on each estrous cycle stage. At 2 mpi, IHKA-L females displayed higher seizure frequency than IHKA-R during diestrus and estrus (diestrus: *t* = 2.05, *p*-value = 0.05; estrus *t* = 2.41, *p*-value = 0.02; Fig. 3*A*), with a trend toward elevated seizure frequency in IHKA-L females during proestrus (*t* = 1.97, *p* = 0.06; Fig. 3*A*). However, seizure duration and percent time in seizures were higher in IHKA-L compared with IHKA-R females only during diestrus [seizure duration: W = 261, *p*-value = 0.04 (Fig. 3*B*); time in seizure: W = 286, *p*-value = 0.004 (Fig. 3*C*)]. These results suggest that there are distinct elevations in seizure parameters in IHKA-L compared with IHKA-R females at 2 mpi, and that these differences are more pronounced during diestrus.

### Left-right asymmetries and effects of estrous cycle stage on seizure burden in females are dampened by four months after injection

**Figure 4.**
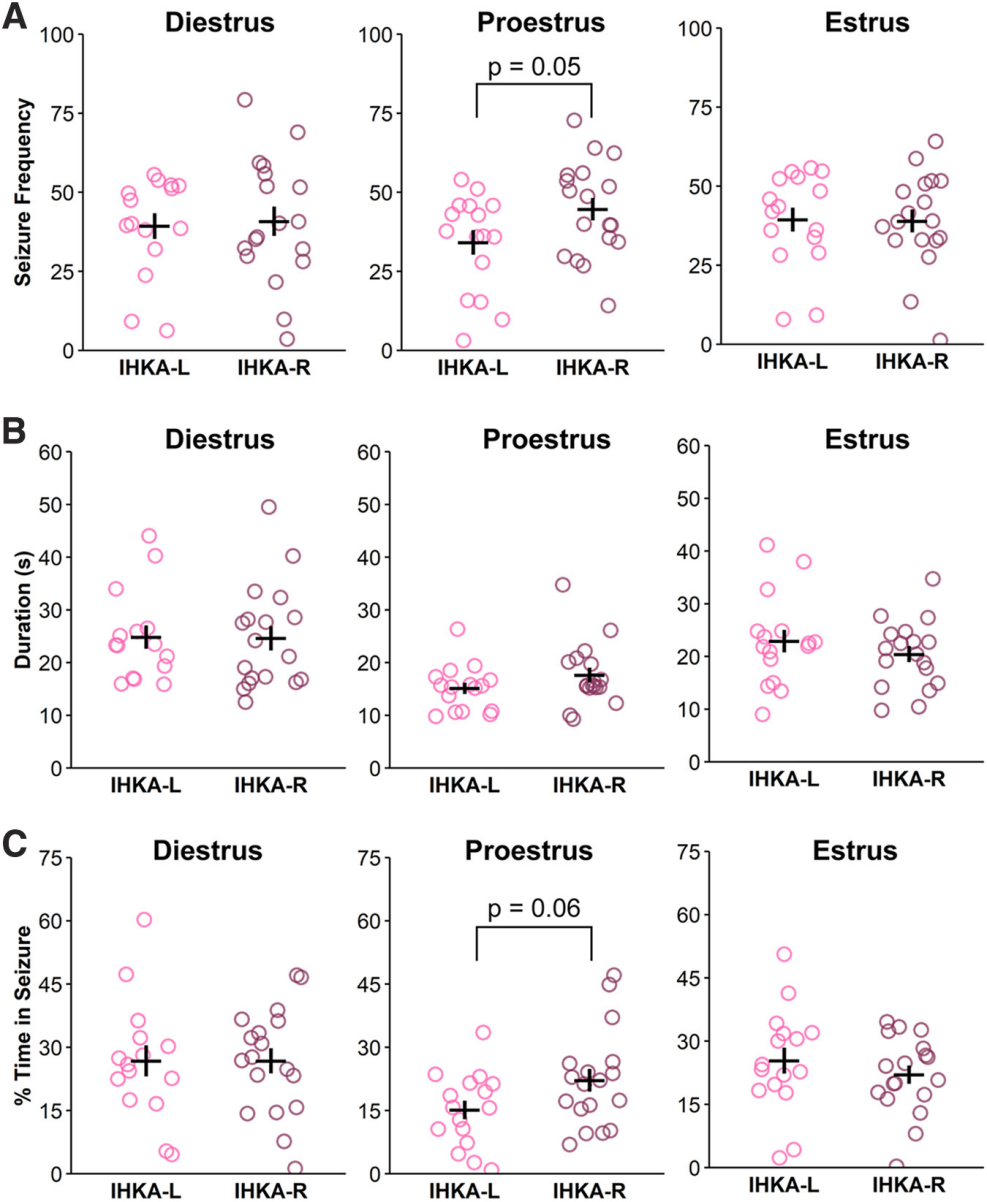
Lateralization and cycle stage effects on seizure burden in females are dampened at four months after IHKA injection. ***A–C***, Circles represent individual values of the average number of seizures per hour (***A***), average seizure duration (***B***), and average percent time in seizure (***C***) for each IHKA female, plotted with mean ± SEM in black lines.

In contrast to the pattern of asymmetric seizure burden at 2 mpi, there were no appreciable differences between IHKA-L and IHKA-R females in any seizure parameters on diestrus or estrus at 4 mpi (Fig. 4). During proestrus, however, IHKA-R animals showed trends for elevated seizure frequency (*t* = −2.00, df = 32, *p* = 0.05; Fig. 4*A*) and percent time in seizures (*t* = −1.99, df = 32, *p* = 0.06; Fig. 4*C*). These results suggest that the influences of injection side on seizure parameters in females are less distinct at 4 mpi.

### Degree of granule cell layer dispersion is not correlated to seizure burden severity

**Figure 5.**
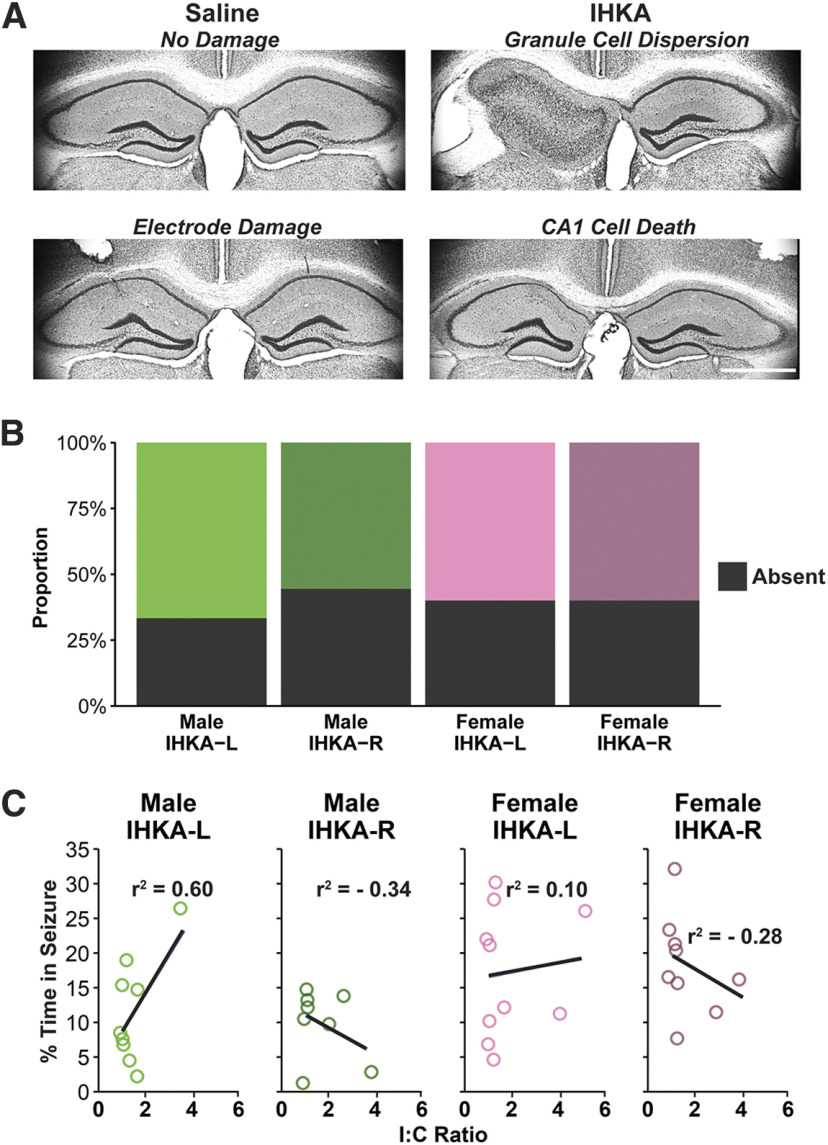
Seizure burden severity is not correlated to the degree of granule cell dispersion at 4 mpi. ***A***, Representative images of Nissl staining, illustrating granule cell dispersion, CA1 cell death, and damage from LFP electrode implantation. Scale bar = 1 mm. ***B***, Proportions of mice in each group that did (colored bars) or did not (black bars) display granule cell dispersion by visual inspection. ***C***, Relationship of the percent time in seizure as a function of the ipsilateral-to-contralateral (I:C) granule cell area ratio. Circles indicate individual mice. Solid lines indicate the linear regression of best fit.

Once LFP recordings were completed, brains from a subset of IHKA-L (male *n* = 9; female *n* = 10) and IHKA-R (male *n* = 8; female *n* = 10) mice were collected and sectioned for analysis of granule cell layer dispersion through visualization with Nissl stain (Fig. 5*A*). Granule cell dispersion was calculated as the ratio between the value for the hippocampus ipsilateral to the site of injection and the equivalent portion of hippocampus contralateral to the side of injection (I:C ratio) (Cutia et al., 2022). There were no differences between IHKA groups in proportions of mice with and without granule cell dispersion (Fig. 5*B*). As all brain samples were collected after completion of recordings at the 4 mpi time point, these ratios were compared with the average percent of time that each animal spent in seizures at the 4 mpi recording period. There were no correlations between percent time spent in seizures and I:C ratio in IHKA-L (males: *r*^2^ = 0.60, *p* = 0.08; females *r*^2^ = 0.1, *p* = 0.77) nor IHKA-R mice (males: *r*^2^ = −0.34, *p* = 0.38; females: *r*^2^ = −0.28, *p* = 0.5; Fig. 5*C*). These results indicate that the degree of granule cell dispersion and seizure severity are not correlated when assessed at 4 mpi in the IHKA model.

### Estrous cycle prolongation is not correlated to seizure burden

**Figure 6.**
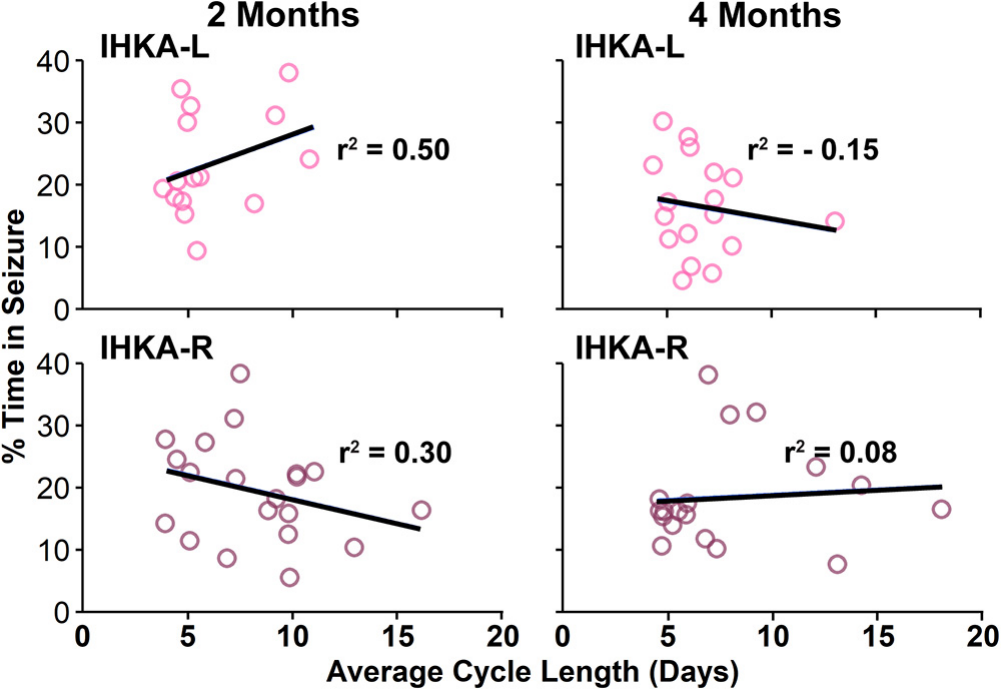
Seizure burden severity is not correlated to the degree of estrous cycle disruption. Percent of time in seizure as a function of estrous cycle length for each mouse evaluated at two (left) and four months postinjection (right) in IHKA-L (top) and IHKA-R (bottom) groups. Circles indicate individual mice. Solid lines indicate the linear regression of best fit.

Estrous cycle disruption is common in a majority of female IHKA mice (J. Li et al., 2017; Cutia et al., 2022). To determine whether the degree of estrous cycle disruption is correlated to the severity of seizure burden, and whether this relationship changes over time, estrous cycle lengths of each mouse were quantified from the cycle monitoring data collected during LFP recordings and correlated to the percentage of time in seizures of each mouse over a 7-d period encompassing all cycle stages. There were no correlations found between estrous cycle length and percent of time in seizures for IHKA-R mice at either 2 (*r*^2^ = 0.3, *p* = 0.20) or 4 mpi (*r*^2^ = 0.08, *p* = 0.75; Fig. 6). IHKA-L females showed a weak correlation at 2 mpi (*r*^2^ = 0.5, *p* = 0.04) but showed no correlation at 4 mpi (*r*^2^ = 0.15, *p* = 0.56). These data align with previous work that reported no significant correlation between cycle length and seizure burden in IHKA-R mice at 2 mpi (J. Li et al., 2020).

### Circulating estradiol and progesterone levels are not correlated with seizure burden at 4 mpi

**Figure 7.**
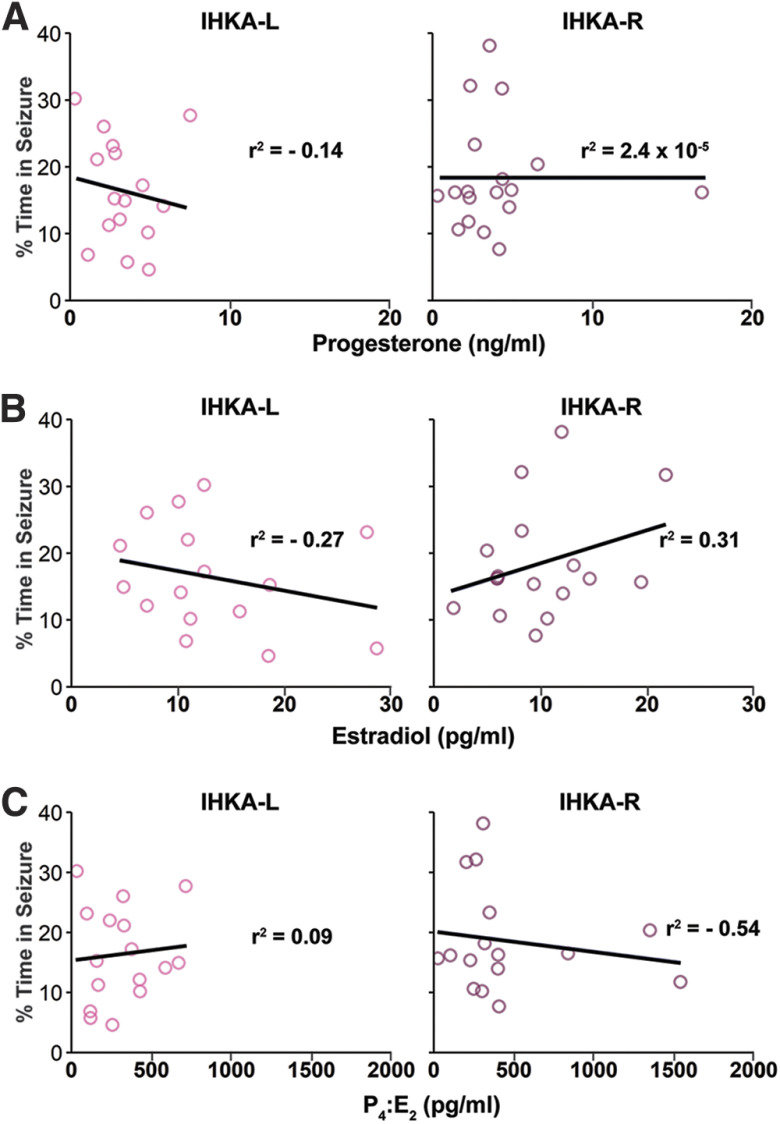
Circulating levels of ovarian hormones on diestrus are not correlated with seizure burden severity at 4 mpi. ***A–C***, Percentage of time spent in seizures as a function of serum progesterone (***A***), estradiol (***B***), and P_4_:E_2_ ratio (***C***) levels of hormones in circulation for each mouse in IHKA-L (left) and IHKA-R (right) groups. Circles indicate individual mice. Solid lines indicate the linear regression of best fit.

Circulating ovarian hormones fluctuate in rodents across the estrous cycle, and seizure activity can also change with estrous cycle stage (Nilsson et al., 2015; Christian et al., 2020). To evaluate whether the chronic seizure burden correlated with circulating ovarian hormone levels in the present cohort, we measured estradiol and progesterone in serum collected on diestrus from the IHKA female animals following completion of LFP recordings at 4 mpi. There were no differences in estradiol (IHKA-L 13.23 ± 1.79 pg/ml, *n* = 16; IHKA-R 10.00 ± 1.27 pg/ml, *n* = 17, *p* = 0.2) or progesterone (IHKA-L 3.36 ± 0.44 ng/ml, *n* = 16; IHKA-R 4.04 ± 0.84 ng/ml, *n* = 18, *p* = 0.86) levels based on the side of IHKA injection. Additionally, there was no difference in the P_4_:E_2_ ratio between IHKA-L (315.20 ± 52.04 pg/ml) and IHKA-R groups (455.58 ± 106.45 pg/ml, *p* = 0.56), consistent with previous findings (Cutia et al., 2022). Furthermore, there was no correlation between circulating progesterone levels and the percent time in seizure in the 7 d before euthanasia for IHKA-L (*r*^2^ = −0.14, *p* = 0.60) or IHKA-R females (*r*^2^ = 2.4 × 10^−5^, *p* = 0.99; Fig. 7*A*). There were also no correlations between estradiol (IHKA-L: *r*^2^ = −0.27, *p* = 0.32; IHKA-R: *r*^2^ = 0.31, *p* = 0.23; Fig. 7*B*) or the P_4_:E_2_ ratio (IHKA-L: *r*^2^ = 0.09, *p* = 0.74; IHKA-R: *r*^2^ = −0.54, *p* = 0.17; Fig. 7*C*) and the percent time in seizure.

## Discussion

Clinical evidence indicates that the hemisphere of a temporal lobe seizure focus may influence seizure cluster patterning in women with epilepsy (Herzog, 2008; Quigg et al., 2009). Preclinical evidence suggests that the IHKA model of TLE holds validity for investigating mechanisms of the aforementioned clinical findings, as differences in hippocampal granule cell dispersion and pituitary gene expression are altered based on site of IHKA injection (Cutia et al., 2022). Despite this evidence, the relationship between seizure patterns in IHKA animals and the site of injection has yet to be documented. In the present study, we evaluated the development and presentation of seizures recorded with LFP at 2 and 4 mpi in male and female IHKA-L and IHKA-R mice. The results indicate a distinct sex difference in seizure parameters between male and female IHKA mice, with higher seizure burden in females. In addition, IHKA-L females display higher overall seizure burden compared with IHKA-R females on diestrus at 2 mpi. On proestrus and estrus, however, this effect is only present for seizure frequency. These results suggest that the phase of the estrous cycle influences left-right asymmetry in seizure burden in IHKA females.

Sex differences have been observed across multiple animal models of TLE and seizure induction (Christian et al., 2020). For example, female rats typically have longer latency to acute seizures and lower mortality rates after systemic pilocarpine injection (Mejías-Aponte et al., 2002). In another study, male rats treated systemically with pilocarpine developed longer lasting seizures over a period of several months, whereas female rats tended to have higher frequency of seizures (Matovu and Cavalheiro, 2022). In a systemic kainic acid injection seizure induction model, male rats had greater susceptibility to seizures than their female counterparts (Mejías-Aponte et al., 2002). In mice, however, the results regarding systemic KA injection have been mixed, with one study reporting higher mortality rates, more severe seizures, and increased neurodegeneration in females (Zhang et al., 2008), but another study describing higher mortality, seizure severity, cognitive impairment, hippocampal neuron loss, and reactive gliosis in males (F. Li and Liu, 2019). With respect to chronic epilepsy in the IHKA model, one report indicated that female mice do not show the same latent period duration following IHKA injection as males, and that hippocampal paroxysmal discharges were rare in females when examined four weeks after injection (Twele et al., 2016). By contrast, another study conducted using the IHKA model did not indicate an effect of sex on seizures, cognitive impairment, or histopathology (Zeidler et al., 2018), although this latter study was not powered to evaluate sex differences. Here, males showed reduced overall seizure burden compared with females, in contrast to other IHKA studies in mice. However, the effect of sex in animal models of epilepsy can vary greatly, influenced by factors such as methodology of seizure induction, measurement criteria for seizures, the species and/or strain of animal, and the age at which seizures are induced (Scharfman and MacLusky, 2014; Christian et al., 2020).

Once brain tissue was harvested at 4 mpi, the level of granule cell dispersion was quantified and correlated to the percentage of time spent in seizures for each animal. The lack of correlations observed between granule cell dispersion and seizure burden is in agreement with other recent studies (Lisgaras and Scharfman, 2022). Although pyramidal cell layer length has been correlated with seizure severity in IHKA mice, this relationship was shown in a cohort in which seizure activity was monitored at the cortical surface using subdural screw electrodes (Lisgaras and Scharfman, 2022). A similar analysis could not be done using the present dataset because of the location of the LFP electrode in the hippocampus. Another limitation is that the tissue could only be collected after all LFP recordings were complete at 4 mpi. In this regard, lateralized differences in granule cell dispersion at 2 mpi were previously shown in a cohort of animals that were not implanted with LFP electrodes (Cutia et al., 2022). As we observed a decrease in lateralized effects in seizure parameters at 4 mpi, it remains possible that granule cell dispersion (and/or other signs of hippocampal sclerosis) and seizure burden are correlated at 2 mpi.

The differences in seizure patterns of IHKA-L and IHKA-R mice on certain estrous cycle stages may arise because of interactions between fluctuating ovarian hormones and basal differences in the left and right hippocampi. For example, the left and right murine hippocampi have distinct populations of synapses (Shinohara et al., 2008; El-Gaby et al., 2015), and there is evidence for left-right asymmetry in hippocampal function in mice (Shipton et al., 2014). There is also evidence that gonadal hormones can impact hippocampal lateralization, with androgen receptor signaling suggested to underlie larger granule cell layer volume in the right hippocampus of both male and female C57BL/6J mice (Tabibnia et al., 1999). Therefore, it is possible that the fluctuations in ovarian hormones across the estrous cycle, and lateralized interactions between ovarian hormones and the hippocampus, produce left-right asymmetry in emergent hippocampal phenotypes. In mice, the P_4_:E_2_ ratio is higher on diestrus than on proestrus and estrus (Walmer et al., 1992; Christian et al., 2020), and lower P_4_:E_2_ may promote seizure activity in humans (Bonuccelli et al., 1989; Herzog et al., 1997; Herzog, 2015). A limitation of the lack of correlation between chronic seizure burden and progesterone/estradiol levels in the present study is that the hormones were measured in blood samples collected at 4 mpi, whereas the left-right asymmetry in seizure burden was most prominent at 2 mpi. However, previous work indicated a lack of difference in serum progesterone and estradiol, and P_4_:E_2_ ratio, between IHKA-L and IHKA-R diestrous females at 2 mpi (Cutia et al., 2022), suggesting that a relationship between circulating levels of these hormones and left-right asymmetry in seizure burden at 2 mpi is unlikely. However, a differential response of left versus right hippocampus to estradiol and/or progesterone actions cannot be ruled out, nor can differences in local hippocampal synthesis of these hormones or their metabolites (Tokuda et al., 2011; Sato and Woolley, 2016; Brann et al., 2022).

Previous work established that the estrous cycle can have impacts on interictal spike presentation in systemic kainic acid and pilocarpine models of TLE in rats (D’Amour et al., 2015), and IHKA mice can exhibit elevated seizure duration and time spent in seizures on proestrus and estrus combined compared with diestrus (J. Li et al., 2020). Importantly, most IHKA female mice exhibit elongated estrous cycles (J. Li et al., 2017, 2018, 2020; Cutia et al., 2022; Ingram et al., 2022; J. Li and Christian-Hinman, 2022). Here, estrous cycle length was not significantly correlated to seizure burden in either IHKA-L or -R mice, consistent with previous reports (J. Li et al., 2020). Therefore, it does not appear that higher epilepsy severity drives greater estrous cycle disruption, nor is there a reciprocal relationship of cycle disruption promoting increased seizure burden in the IHKA model. In previous work examining mice without LFP recordings, estrous cycle disruption persisted to 4 mpi in IHKA-R but not IHKA-L females (Cutia et al., 2022). The dissipation in phenotypes in IHKA-L females suggests that asymmetries may be influenced by the progression of the disease, the increased age of the animals, or both. The progressive decline of certain physiological functions as mice age could shape the varied patterns seen at different timepoints. It is also possible that the observed differences at 2 mpi are indicative of differential rates of disease progression that eventually normalize, such that seizure severity lacks overt sex-based and hemisphere-based differences by 4 mpi. In total, these findings suggest that injection site and time of recording after injection in IHKA female mice are factors to be considered in experimental design that can shape overall seizure burden outcomes.

The female-specific presence of seizure burden asymmetries aligns with clinical findings of reproductive endocrine disorder development among TLE patients. Male and female patients with TLE often develop reproductive endocrine dysfunction (Herzog et al., 1986a, b), but females more consistently develop higher rates of dysfunction than the general population (Herzog et al., 1986b; Herzog and Schachter, 2001). Moreover, the propensities for developing polycystic ovary syndrome or hypothalamic amenorrhea are differentially based on the hemisphere of the seizure focus in females (Herzog, 1993; Kalinin and Zheleznova, 2007). These findings indicate that there may be differential mechanisms in the left and right hemispheres that contribute to the ability of seizures to promote reproductive endocrine dysfunction. Furthermore, the different patterns of seizure clustering in association with menstrual cycle phase in females with left-sided or right-sided TLE (Herzog, 2008; Quigg et al., 2009) suggest that left-right asymmetry can shape the presentation of both epilepsy and associated comorbidities. The present findings of female-specific asymmetry in seizure presentation are thus consistent with clinical reports, and support the translational validity of the IHKA mouse model in recapitulating at least some mechanistic differences in structural or functional changes because of seizures in the left and right hippocampus, as well as sex differences in this lateralization.

The IHKA mouse model displays many characteristics recapitulating human epilepsy, underscoring the validity of its use in investigating neural mechanisms of TLE. The present work indicates that seizure burden outcomes within this preclinical model are influenced by sex and, in females, laterality of epileptogenic insult. Female mice also show estrous cycle-associated changes in seizure activity that vary depending on the hemisphere of targeted hippocampal damage. This work thus reveals female-specific left-right asymmetry in functional properties of the murine hippocampus, with implications for both normal and disease states.

## Synthesis

Reviewing Editor: Viji Santhakumar, University of California Riverside Decisions are customarily a result of the Reviewing Editor and the peer reviewers coming together and discussing their recommendations until a consensus is reached. When revisions are invited, a fact-based synthesis statement explaining their decision and outlining what is needed to prepare a revision will be listed below. The following reviewer(s) agreed to reveal their identity: Anastasia Brodovskaya.

The study examines how sex and lateralization of IHKA injection, a model of epilepsy, impacts seizure frequency/duration. They find that females have greater seizure frequency/duration than males 2 month after injection the difference in duration maintained at 4 months. Females but not males showed lateralization with IHKA-L female mice exhibiting higher seizure frequency on diestrus and estrus at 2 months post-injection than IHKA-R female mice. The manuscript is clear, easy to follow and well designed. The sample size is adequate at several time points to gather the seizure frequency, duration, and time in seizure data. Investigation of the impact of the estrous cycle stage on seizure initiation has not been previously examined and is a relevant question for the field.

1. Since there is prior literature demonstrating that some female mice have disruption of their estrous cycle (Jiang Li et al. 2016, Epilepsia Open), it does not seem appropriate to pool these mice. The authors should determine if there are differences between female mice with short vs long cycle?

2. What is the progesterone and estrogen levels on diestrus and estrus? The hormones circulate in the blood, but the authors show that the left and right hippocampus respond differently. If the authors are attributing such differences to hormonal levels, it would be ideal to determine intrahippocampal progesterone and estrogen levels from the left and right hippocampal tissue. However, the authors should at least provide blood hormone levels

3. The “seizure traces” in Fig 1A lack the resolution to confidently distinguished between seizures or noise. Seizure characteristics including spike-and-wave discharges and postictal suppression and morphology are impossible to identify with the current resolution. The authors should expand the view of Fig 1A and show traces with at least 10 s resolution and characteristic spike-and-wave discharges.

4. Fig. 3A shows that some mice on diestrus had up to 100 seizures per hour. If the average seizure lasted 20 s, doesn’t it meet the definition of status epilepticus (20% of 1 hr is spent seizing)? Could the mice with status epilepticus be pooled with mice that had seizures?

5. The first part of the 4th paragraph that reviews the reorganization across the menstrual cycle in humans needs to be better organized. The first part is more descriptive of menstrual cycle and function ramifications then the topic switches to the differences in estrous cycle and seizure progression. This link is difficult to follow and since no histological measurements were made in the study the comparison goes beyond the data. The link seems tenuous and distracts from the issue of endocrine changes in TLE in humans compared to mice. There are too many points made in the paragraph and needs to be clarified.

6. A caveat to the study is the lack of histological validation of injury site. This should be discussed.

7. The authors do not provide the number of animals used for each experiment. Does each point on the graph represent an individual mouse? The authors need to illustrate SEM, in addition to mean, for each graph. Inclusion of estimation statistics is recommended.

8. Please provide a citation for the first sentence of Discussion.

9. The authors discuss that disease progression may account for the difference in seizure duration between 2 and 4 months. In this regard, it would be helpful to describe what is known about disease progression in IHKA model. Is there a difference in neuronal death and gliosis? It might be that the sex and hemisphere differences at 2 mpi may signal more progression of the disease that tends to plateau by 4 months of age so the difference in seizure severity are no longer detected at 4 mpi. This discussion may be helpful to understand the effect of time after seizure induction in their study.

10. It would be helpful to define what perimenstrual catamenial seizures are in the introduction.

11. Methods: Please indicate whether mice were singly housed or group housed.

## Author Response

Authors’ note: We thank the reviewers for their helpful comments, which we have addressed to improve the presentation and discussion of our work. To address the reviewers’ concerns, we have added new histological analyses, new measurements of circulating estradiol and progesterone, new analysis of the relationship between estrous cycle length and seizure parameters, and a new table describing the estimation statistics used. We have also extensively revised the Introduction and Discussion and added new references to improve clarity, incorporate the important points raised by the reviewers, and improve flow and readability.

Reviewer Comments: The study examines how sex and lateralization of IHKA injection, a model of epilepsy, impacts seizure frequency/duration. They find that females have greater seizure frequency/duration than males 2 month after injection the difference in duration maintained at 4 months. Females but not males showed lateralization with IHKA-L female mice exhibiting higher seizure frequency on diestrus and estrus at 2 months post-injection than IHKA-R female mice. The manuscript is clear, easy to follow and well designed. The sample size is adequate at several time points to gather the seizure frequency, duration, and time in seizure data. Investigation of the impact of the estrous cycle stage on seizure initiation has not been previously examined and is a relevant question for the field.

1. Since there is prior literature demonstrating that some female mice have disruption of their estrous cycle (Jiang Li et al. 2016, Epilepsia Open), it does not seem appropriate to pool these mice. The authors should determine if there are differences between female mice with short vs long cycle?

Response: This is an important point. Despite the development of disrupted estrous cycles, it has been previously established that the degree of estrous cycle disruption is not related to epilepsy severity in the IHKA mouse model (Li et al., 2020). To further determine whether this lack of a relationship is also observed in this study, we performed new analyses and added new Figure 6 examining linear correlation statistics between average estrous cycle length and the percent time spent in seizure. We found a weak correlation (p=0.04) for the IHKA-L group at 2 mpi, but the other groups all showed a lack of correlation. In addition to the new Figure 6, we have edited the Methods, Results, and Discussion to incorporate these new analyses.

2. What is the progesterone and estrogen levels on diestrus and estrus? The hormones circulate in the blood, but the authors show that the left and right hippocampus respond differently. If the authors are attributing such differences to hormonal levels, it would be ideal to determine intrahippocampal progesterone and estrogen levels from the left and right hippocampal tissue. However, the authors should at least provide blood hormone levels.

Response: Although we cannot discern relationships between LFP recordings at 2 mpi and circulating hormones due to the nature of the blood/serum collection in this study, we have evaluated the circulating progesterone and estradiol levels at 4 mpi from samples collected on diestrus. The manuscript has been updated the reflect the addition of such data in new Figure 7.

We agree that direct measurement of intrahippocampal values of progesterone and estradiol would be informative, but the tissue was not collected in a manner that would allow for this assessment; we have noted this as a point of future investigation in the discussion. We have edited the Methods, Results, and Discussion to incorporate these new experiments and data.

3. The “seizure traces” in Fig 1A lack the resolution to confidently distinguished between seizures or noise. Seizure characteristics including spike-and-wave discharges and postictal suppression and morphology are impossible to identify with the current resolution. The authors 2 should expand the view of Fig 1A and show traces with at least 10 s resolution and characteristic spike-and-wave discharges.

Response: We have updated the traces in Figure 1 to appear at a resolution of 10 s to assist in identification of discharges present in the LFP recordings. We thank the reviewers for this suggestion.

4. Fig. 3A shows that some mice on diestrus had up to 100 seizures per hour. If the average seizure lasted 20 s, doesn’t it meet the definition of status epilepticus (20% of 1 hr is spent seizing)? Could the mice with status epilepticus be pooled with mice that had seizures?

Response: This is an important point, and we thank the reviewer for highlighting that some of the mice do indeed spend a large amount of time in seizures and that there is high variability in the IHKA model. To determine whether the results are skewed by including mice with severe epilepsy, using the suggested cutoff of 20% time in seizures, we performed a new analysis, now only comparing mice in each group that spent less than 20% of time in seizures. In this new analysis, IHKA-L mice spent more time in seizures than IHKA-R mice on diestrus (p = 0.04), but not on other estrous cycle stages. Therefore, the same lateralization effects are found in this subset as when all mice included, regardless of seizure burden. It should be noted that these events are almost entirely electrographic seizures and have little to no behavioral manifestations, as commonly observed in the IHKA mouse model (Bouilleret et al., 1999; Groticke et al., 2008; Klein et al., 2015). Additionally, the frequency of seizures seen in our animals is in line with other work conducted at chronic timepoints in the IHKA model (Klein et al., Neuropharmacology, 2015, Twele et al., Neuropharmacology, 2015). We have edited he

Methods to note this aspect of the IHKA model.

5. The first part of the 4th paragraph that reviews the reorganization across the menstrual cycle in humans needs to be better organized. The first part is more descriptive of menstrual cycle and function ramifications then the topic switches to the differences in estrous cycle and seizure progression. This link is difficult to follow and since no histological measurements were made in the study the comparison goes beyond the data. The link seems tenuous and distracts from the issue of endocrine changes in TLE in humans compared to mice. There are too many points made in the paragraph and needs to be clarified.

Response: We thank the reviewer for the suggestion to aid in clarity. The description of the human studies has been condensed and moved to the Introduction, and the aforementioned paragraph has been reorganized.

6. A caveat to the study is the lack of histological validation of injury site. This should be discussed.

Response: We have added a quantification of the granule cell layer dispersion values as a ratio of the areas of the contralateral to ipsilateral hippocampal regions, and found no correlations between the degree of granule cell dispersion and seizure burden. This is similar to recent results from other laboratories (Lisgaras & Scharfman, 2022). Although pyramidal cell layer length has been correlated to seizure burden (Lisgaras & Scharfman, 2022) such measures could not be accurately reported in the present animals due to damage caused by depth electrode. We have added the granule cell dispersion data as new Figure 5, and have edited the Methods, Results, and Discussion accordingly.

7. The authors do not provide the number of animals used for each experiment. Does each 3 point on the graph represent an individual mouse? The authors need to illustrate SEM, in addition to mean, for each graph. Inclusion of estimation statistics is recommended.

Response: The numbers of mice in LFP recordings, separated by injection site and sex, were previously provided in the first sentence of the Results section, and the loss of two IHKA-R mice prior to the 4-month time point was already noted. Numbers of mice used in new analyses of histology and hormone assays are now noted in the appropriate Results sections. The figure legends previously stated that the points on each graph were “individual values,” but legends have been edited to “Circles represent individual values... for each mouse” (and similar depending on the figure) for clarity. Mean {plus minus} SEM was already included in the previous versions of the figures, and as noted in the legends, but we have added, “in black lines,” for clarity. We have added new Table 1 to provide information on the estimation statistics for the significant results in this study.

8. Please provide a citation for the first sentence of Discussion.

Response: We thank the reviewer for catching this error. The text has been updated accordingly.

9. The authors discuss that disease progression may account for the difference in seizure duration between 2 and 4 months. In this regard, it would be helpful to describe what is known about disease progression in IHKA model. Is there a difference in neuronal death and gliosis? It might be that the sex and hemisphere differences at 2 mpi may signal more progression of the disease that tends to plateau by 4 months of age so the difference in seizure severity are no longer detected at 4 mpi. This discussion may be helpful to understand the effect of time after seizure induction in their study.

Response: Although there have been reports that seizures in the IHKA mouse model occur many months after injection (Bouilleret et al., 1999; Bui et al., 2018), data beyond 2 months post-injection are limited (Henshall, 2017). It is also possible that the observed differences at the 2 mpi timepoint are indicative of differences in disease progression, and that a plateau in seizure severity by 4 mpi results in a lack of sex and hemisphere differences. We have added a sentence to this effect in the Discussion.

10. It would be helpful to define what perimenstrual catamenial seizures are in the introduction.

Response: We thank the reviewer for suggesting this point of clarification. The text of the

Introduction has been updated accordingly.

11. Methods: Please indicate whether mice were singly housed or group housed.

Response: The last sentence of the Stereotaxic IHKA and LFP electrode implantation surgeries section of the Methods already stated that, “Mice were singly housed after electrode implantation and for the duration of the remaining experimental period.” However, for added clarification, we have added a further statement regarding the change from group housing to single housing after electrode implantation to the first paragraph of the Methods.

## References

[B1] Addis DR, Moscovitch M, McAndrews MP (2007) Consequences of hippocampal damage across the autobiographical memory network in left temporal lobe epilepsy. Brain 130:2327–2342. 10.1093/brain/awm166 17681983

[B2] Alessio A, Bonilha L, Rorden C, Kobayashi E, Min LL, Damasceno BP, Cendes F (2006) Memory and language impairments and their relationships to hippocampal and perirhinal cortex damage in patients with medial temporal lobe epilepsy. Epilepsy Behav 8:593–600. 10.1016/j.yebeh.2006.01.007 16517214

[B3] Armstrong C, Krook-Magnuson E, Oijala M, Soltesz I (2013) Closed-loop optogenetic intervention in mice. Nat Protoc 8:1475–1493. 10.1038/nprot.2013.080 23845961 PMC3988315

[B4] Bonuccelli U, Melis GB, Paoletti AM, Fioretti P, Murri L, Muratorio A (1989) Unbalanced progesterone and estradiol secretion in catamenial epilepsy. Epilepsy Res 3:100–106. 10.1016/0920-1211(89)90037-5 2651113

[B5] Bouilleret V, Ridoux V, Depaulis A, Marescaux C, Nehlig A, Le Gal La Salle G (1999) Recurrent seizures and hippocampal sclerosis following intrahippocampal kainate injection in adult mice: electroencephalography, histopathology and synaptic reorganization similar to mesial temporal lobe epilepsy. Neuroscience 89:717–729. 10.1016/s0306-4522(98)00401-1 10199607

[B6] Brann DW, Lu Y, Wang J, Zhang Q, Thakkar R, Sareddy GR, Pratap UP, Tekmal RR, Vadlamudi RK (2022) Brain-derived estrogen and neural function. Neurosci Biobehav Rev 132:793–817. 10.1016/j.neubiorev.2021.11.014 34823913 PMC8816863

[B7] Choi H, Detyniecki K, Bazil C, Thornton S, Crosta P, Tolba H, Muneeb M, Hirsch LJ, Heinzen EL, Sen A, Depondt C, Perucca P, Heiman GA; EPIGEN Consortium (2020) Development and validation of a predictive model of drug-resistant genetic generalized epilepsy. Neurology 95:e2150–e2160. 10.1212/WNL.0000000000010597 32759205 PMC7713754

[B8] Christian CA, Reddy DS, Maguire J, Forcelli PA (2020) Sex differences in the epilepsies and associated comorbidities: implications for use and development of pharmacotherapies. Pharmacol Rev 72:767–800. 10.1124/pr.119.017392 32817274 PMC7495340

[B9] Cutia CA, Leverton LK, Ge X, Youssef R, Raetzman LT, Christian-Hinman CA (2022) Phenotypic differences based on lateralization of intrahippocampal kainic acid injection in female mice. Exp Neurol 355:114118. 10.1016/j.expneurol.2022.114118 35597270 PMC10462257

[B10] D’Amour J, Magagna-Poveda A, Moretto J, Friedman D, LaFrancois JJ, Pearce P, Fenton AA, MacLusky NJ, Scharfman HE (2015) Interictal spike frequency varies with ovarian cycle stage in a rat model of epilepsy. Exp Neurol 269:102–119. 10.1016/j.expneurol.2015.04.003 25864929 PMC4446145

[B11] El-Gaby M, Shipton OA, Paulsen O (2015) Synaptic plasticity and memory: new insights from hippocampal left–right asymmetries. Neuroscientist 21:490–502. 10.1177/1073858414550658 25239943

[B12] Engel J (2001) Mesial temporal lobe epilepsy: what have we learned? Neuroscientist 7:340–352. 10.1177/107385840100700410 11488399

[B13] French JA, Williamson PD, Thadani VM, Darcey TM, Mattson RH, Spencer SS, Spencer DD (1993) Characteristics of medial temporal lobe epilepsy: I. Results of history and physical examination. Ann Neurol 34:774–780. 10.1002/ana.410340604 8250525

[B14] Gazzaniga MS (1995) Principles of human brain organization derived from split-brain studies. Neuron 14:217–228. 10.1016/0896-6273(95)90280-5 7857634

[B15] Gloe LM, Kashy DA, Jacobs EG, Klump KL, Moser JS (2021) Examining the role of ovarian hormones in the association between worry and working memory across the menstrual cycle. Psychoneuroendocrinology 131:105285. 10.1016/j.psyneuen.2021.105285 34090137 PMC8405555

[B16] Gröticke I, Hoffmann K, Löscher W (2008) Behavioral alterations in a mouse model of temporal lobe epilepsy induced by intrahippocampal injection of kainate. Exp Neurol 213:71–83. 10.1016/j.expneurol.2008.04.036 18585709

[B17] Herzog AG (1993) A relationship between particular reproductive endocrine disorders and the laterality of epileptiform discharges in women with epilepsy. Neurology 43:1907–1910. 10.1212/wnl.43.10.1907 8413946

[B18] Herzog AG (2008) Catamenial epilepsy: definition, prevalence pathophysiology and treatment. Seizure 17:151–159. 10.1016/j.seizure.2007.11.014 18164632

[B19] Herzog AG (2015) Catamenial epilepsy: update on prevalence, pathophysiology and treatment from the findings of the NIH Progesterone Treatment Trial. Seizure 28:18–25. 10.1016/j.seizure.2015.02.024 25770028

[B20] Herzog AG, Schachter SC (2001) Valproate and the polycystic ovarian syndrome: final thoughts. Epilepsia 42:311–315. 10.1046/j.1528-1157.2001.33500.x 11442145

[B21] Herzog AG, Klein P, Ransil BJ (1997) Three patterns of catamenial epilepsy. Epilepsia 38:1082–1088. 10.1111/j.1528-1157.1997.tb01197.x 9579954

[B22] Herzog AG, Seibel MM, Schomer DL, Vaitukaitis JL, Geschwind N (1986a) Reproductive endocrine disorders in men with partial seizures of temporal lobe origin. Arch Neurol 43:347–350. 10.1001/archneur.1986.00520040035015 3082313

[B23] Herzog AG, Seibel MM, Schomer DL, Vaitukaitis JL, Geschwind N (1986b) Reproductive endocrine disorders in women with partial seizures of temporal lobe origin. Arch Neurol 43:341–346. 10.1001/archneur.1986.00520040029014 2937394

[B24] Howard LR, Kumaran D, Ólafsdóttir HF, Spiers HJ (2011) Double dissociation between hippocampal and parahippocampal responses to object–background context and scene novelty. J Neurosci 31:5253–5261. 10.1523/JNEUROSCI.6055-10.2011 21471360 PMC3079899

[B25] Ingram RJ, Leverton LK, Daniels VC, Li J, Christian-Hinman CA (2022) Increased GABA transmission to GnRH neurons after intrahippocampal kainic acid injection in mice is sex-specific and associated with estrous cycle disruption. Neurobiol Dis 172:105822. 10.1016/j.nbd.2022.105822 35868435 PMC9455811

[B26] Jordan JT (2020) The rodent hippocampus as a bilateral structure: a review of hemispheric lateralization. Hippocampus 30:278–292. 10.1002/hipo.23188 31880377

[B27] Kalinin VV, Zheleznova EV (2007) Chronology and evolution of temporal lobe epilepsy and endocrine reproductive dysfunction in women: relationships to side of focus and catameniality. Epilepsy Behav 11:185–191. 10.1016/j.yebeh.2007.04.014 17573242

[B28] Klein S, Bankstahl M, Löscher W (2015) Inter-individual variation in the effect of antiepileptic drugs in the intrahippocampal kainate model of mesial temporal lobe epilepsy in mice. Neuropharmacology 90:53–62. 10.1016/j.neuropharm.2014.11.008 25460186

[B29] Li F, Liu L (2019) Comparison of kainate-induced seizures, cognitive impairment and hippocampal damage in male and female mice. Life Sci 232:116621. 10.1016/j.lfs.2019.116621 31269415

[B30] Li J, Christian-Hinman CA (2022) Epilepsy-associated increase in gonadotropin-releasing hormone neuron firing in diestrous female mice is independent of chronic seizure burden severity. Epilepsy Res 184:106948. 10.1016/j.eplepsyres.2022.106948 35690025 PMC10416707

[B31] Li J, Kim JS, Abejuela VA, Lamano JB, Klein NJ, Christian CA (2017) Disrupted female estrous cyclicity in the intrahippocampal kainic acid mouse model of temporal lobe epilepsy. Epilepsia Open 2:39–47. 10.1002/epi4.12026 29750212 PMC5939433

[B32] Li J, Leverton LK, Naganatanahalli LM, Christian-Hinman CA (2020) Seizure burden fluctuates with the female reproductive cycle in a mouse model of chronic temporal lobe epilepsy. Exp Neurol 334:113492. 10.1016/j.expneurol.2020.113492 33007292 PMC7642196

[B33] Li J, Robare JA, Gao L, Ghane MA, Flaws JA, Nelson ME, Christian CA (2018) Dynamic and sex-specific changes in gonadotropin-releasing hormone neuron activity and excitability in a mouse model of temporal lobe epilepsy. eNeuro 5:ENEURO.0273-18.2018. 10.1523/ENEURO.0273-18.2018 PMC6153338 30255128

[B34] Lisgaras CP, Scharfman HE (2022) Robust chronic convulsive seizures, high frequency oscillations, and human seizure onset patterns in an intrahippocampal kainic acid model in mice. Neurobiol Dis 166:105637. 10.1016/j.nbd.2022.105637 35091040 PMC9034729

[B35] Maguire EA, Frith CD (2003) Lateral asymmetry in the hippocampal response to the remoteness of autobiographical memories. J Neurosci 23:5302–5307. 10.1523/JNEUROSCI.23-12-05302.2003 12832555 PMC6741182

[B36] Mathern GW, Babb TL, Vickrey BG, Melendez M, Pretorius JK (1995) The clinical-pathogenic mechanisms of hippocampal neuron loss and surgical outcomes in temporal lobe epilepsy. Brain 118:105–118. 10.1093/brain/118.1.105 7894997

[B37] Matovu D, Cavalheiro EA (2022) Differences in evolution of epileptic seizures and topographical distribution of tissue damage in selected limbic structures between male and female rats submitted to the pilocarpine model. Front Neurol 13:802587. 10.3389/fneur.2022.802587 35449517 PMC9017681

[B38] Mejías-Aponte CA, Jiménez-Rivera CA, Segarra AC (2002) Sex differences in models of temporal lobe epilepsy: role of testosterone. Brain Res 944:210–218. 10.1016/s0006-8993(02)02691-4 12106683

[B39] Nilsson ME, Vandenput L, Tivesten Å, Norlén A-K, Lagerquist MK, Windahl SH, Börjesson AE, Farman HH, Poutanen M, Benrick A, Maliqueo M, Stener-Victorin E, Ryberg H, Ohlsson C (2015) Measurement of a comprehensive sex steroid profile in rodent serum by high-sensitive gas chromatography-tandem mass spectrometry. Endocrinology 156:2492–2502. 10.1210/en.2014-1890 25856427

[B40] Pantier LK, Li J, Christian CA (2019) Estrous cycle monitoring in mice with rapid data visualization and analysis. Bio Protoc 9:e3354. 10.21769/BioProtoc.3354 PMC7372919 32695847

[B41] Phuong TH, Houot M, Méré M, Denos M, Samson S, Dupont S (2021) Cognitive impairment in temporal lobe epilepsy: contributions of lesion, localization and lateralization. J Neurol 268:1443–1452. 10.1007/s00415-020-10307-6 33216221

[B42] Quigg M, Smithson SD, Fowler KM, Sursal T, Herzog AG; NIH Progesterone Trial Study Group (2009) Laterality and location influence catamenial seizure expression in women with partial epilepsy. Neurology 73:223–227. 10.1212/WNL.0b013e3181ae7adf 19620611 PMC2715574

[B43] Riban V, Bouilleret V, Pham-Lê BT, Fritschy J-M, Marescaux C, Depaulis A (2002) Evolution of hippocampal epileptic activity during the development of hippocampal sclerosis in a mouse model of temporal lobe epilepsy. Neuroscience 112:101–111. 10.1016/s0306-4522(02)00064-7 12044475

[B44] Rusina E, Bernard C, Williamson A (2021) The kainic acid models of temporal lobe epilepsy. eNeuro 8:ENEURO.0337-20.2021. 10.1523/ENEURO.0337-20.2021 PMC8174050 33658312

[B45] Sato SM, Woolley CS (2016) Acute inhibition of neurosteroid estrogen synthesis suppresses status epilepticus in an animal model. Elife 5:e12917. 10.7554/eLife.12917 27083045 PMC4862752

[B46] Schaapsmeerders P, van Uden IWM, Tuladhar AM, Maaijwee NAM, van Dijk EJ, Rutten-Jacobs LCA, Arntz RM, Schoonderwaldt HC, Dorresteijn LDA, de Leeuw F-E, Kessels RPC (2015) Ipsilateral hippocampal atrophy is associated with long-term memory dysfunction after ischemic stroke in young adults. Hum Brain Mapp 36:2432–2442. 10.1002/hbm.22782 25757914 PMC6869088

[B47] Scharfman HE, MacLusky NJ (2014) Sex differences in the neurobiology of epilepsy: a preclinical perspective. Neurobiol Dis 72:180–192. 10.1016/j.nbd.2014.07.004 25058745 PMC4252793

[B48] Shinohara Y, Hirase H, Watanabe M, Itakura M, Takahashi M, Shigemoto R (2008) Left-right asymmetry of the hippocampal synapses with differential subunit allocation of glutamate receptors. Proc Natl Acad Sci U S A 105:19498–19503. 10.1073/pnas.0807461105 19052236 PMC2593619

[B49] Shipton OA, El-Gaby M, Apergis-Schoute J, Deisseroth K, Bannerman DM, Paulsen O, Kohl MM (2014) Left–right dissociation of hippocampal memory processes in mice. Proc Natl Acad Sci U S A 111:15238–15243. 10.1073/pnas.1405648111 25246561 PMC4210314

[B50] Tabibnia G, Cooke BM, Breedlove SM (1999) Sex difference and laterality in the volume of mouse dentate gyrus granule cell layer. Brain Res 827:41–45. 10.1016/s0006-8993(99)01262-7 10320691

[B51] Taylor CM, Pritschet L, Olsen RK, Layher E, Santander T, Grafton ST, Jacobs EG (2020) Progesterone shapes medial temporal lobe volume across the human menstrual cycle. Neuroimage 220:117125. 10.1016/j.neuroimage.2020.117125 32634592

[B52] Tokuda K, Izumi Y, Zorumski CF (2011) Ethanol enhances neurosteroidogenesis in hippocampal pyramidal neurons by paradoxical NMDA receptor activation. J Neurosci 31:9905–9909. 10.1523/JNEUROSCI.1660-11.2011 21734282 PMC3180997

[B53] Twele F, Töllner K, Brandt C, Löscher W (2016) Significant effects of sex, strain, and anesthesia in the intrahippocampal kainate mouse model of mesial temporal lobe epilepsy. Epilepsy Behav 55:47–56. 10.1016/j.yebeh.2015.11.027 26736063

[B54] Walmer DK, Wrona MA, Hughes CL, Nelson KG (1992) Lactoferrin expression in the mouse reproductive tract during the natural estrous cycle: correlation with circulating estradiol and progesterone. Endocrinology 131:1458–1466. 10.1210/endo.131.3.1505477 1505477

[B55] Zeidler Z, Brandt-Fontaine M, Leintz C, Krook-Magnuson C, Netoff T, Krook-Magnuson E (2018) Targeting the mouse ventral hippocampus in the intrahippocampal kainic acid model of temporal lobe epilepsy. eNeuro 5:ENEURO.0158-18.2018. 10.1523/ENEURO.0158-18.2018 PMC6102375 30131968

[B56] Zhang XM, Zhu SW, Duan RS, Mohammed AH, Winblad B, Zhu J (2008) Gender differences in susceptibility to kainic acid-induced neurodegeneration in aged C57BL/6 mice. Neurotoxicology 29:406–412. 10.1016/j.neuro.2008.01.006 18342945

